# The Radical Treatment of the Maxillary Antrum

**Published:** 1905-03

**Authors:** Lee Maidment Hurd


### THE RADICAL TREATMENT OF THE MAXILLARY ANTRUM.[1]

BY LEE MAIDMENT HURD, M.D.[2]

THE antrum of Highmore, as we all know, is liable to become the seat of a suppurative inflammation either acute or chronic. Of the chronic, which we are about to consider, nearly one-half are secondary to an inflammatory condition about the roots of the teeth, and practically all the others are of nasal origin. The chronic cases may begin with an acute inflammation which subsides into a chronic condition, or it may be insidious from the beginning.

The more prominent symptoms are a purulent discharge from the nose, sometimes so slight as hardly to be noticeable; in fact, the most frequent cause of one-sided purulent nasal discharge is antrum disease. Nasal obstruction on the affected side, sometimes on both sides, is frequent. Pain around the eye is found in a great many cases, but pain in or over the antrum is rare and is only caused by retention of the pus under pressure or a neurasthenic tendency in the patient. Trigeminal neuralgia, pain in the vertex or occiput, are frequently present when the sphenoid is involved, and pain to the inner side of the eye generally means that the anterior ethmoid cells are affected. Tenderness on pressure over the canine fossa is generally present, but not invariably. Gastric disturbances are frequently present from the swallowing of pus at night; also a bad taste in the mouth in the morning. Sometimes frontal headaches are present, but more frequently infraorbital neuralgia or shooting pains in the teeth. This dentalgia does not necessarily mean that the antral trouble originated in the teeth. The pus in the nose sooner or later sets up a chronic rhinitis. Once in a great while eye symptoms develop, affections of the tear ducts, a recurring iritis, or a chorioretinitis.

In regard to diagnosis, pus under the middle turbinate may come from the frontal or anterior ethmoid cells as well as from the

---

[1] Read before The New York Institute of Stomatology, December 6, 1904.

[2] Assistant Surgeon, Manhattan Eye and Ear Hospital.

antrum. As the antral orifice is near the roof, the head held horizontally and to the opposite side will increase the flow of pus, if it is not too viscid. A small silver canula can sometimes be passed through the antral orifice and the cavity either inflated with compressed air or irrigated, which will bring out whatever it contains and give valuable aid in arriving at a diagnosis. It is justfiable to remove part of the middle turbinate to gain an entrance to the antral orifice. If you still fail, an exploratory puncture through the wall of the middle or inferior meatus or through the canine fossa may be made. This is done with a trocar and canula or an especially devised steel exploratory needle. In using the middle meatus route care should be taken not to enter the orbit by mistake.

I prefer the canine fossa or inferior meatus routes, for then one can inflate with compressed air and drive the contents of the antrum out through the normal opening. Aspirating is not very reliable, as the membrane is liable to be quite thick and the needle may not pass through it; and again, the pus may be so viscid that it will not pass through the needle; but by inflating or douching through the needle or canula you will break through whatever membrane may cover it and drive the thick secretion out through the normal orifice, taking for granted that it is open because of the pus in the middle meatus.

Transillumination is of some help, especially where the affected side is quite dark, but is not at all reliable. It may be a rudimentary antrum that causes the darkness, for example.

If there is an opening through a tooth socket from which pus is flowing, then there is not much doubt about the diagnosis.

The hardest part of all is to determine whether the antrum is truly the site of the inflammation or only a reservoir for pus coming down from the frontal or ethmoid cells. This is at times a difficult point to decide, which may require repeated examinations and sometimes treatment of the frontal or ethmoid cells.

In treating the antrum, most of the cases of nasal origin get well spontaneously or with nasal douching. Those of dental origin, as a rule, do not, but treatment of the roots of the affected tooth and the necrotic bone about them, with irrigations through a tooth socket (Cowper's operation) or through the canine fossa, will cure a large per cent. of them. If this method is not successful at the end of three or four months, it never will be. Of the chronic cases, very rarely will a case recover with nasal irrigations alone.

A certain number will be cured by irrigations through a tooth socket. Of those of nasal origin, part will get well by making as large an opening as possible into the antrum through the middle or inferior meatus (Mikulicz's operation), with irrigations and the application of antiseptics. But there are a number of cases in which none of these procedures will effect a cure, and a more radical operation must be performed.

Caldwell, Luc, Boenninghaus, and Jansen at about the same time began to adopt more radical measures. The operation I am about to describe follows the method of Jansen nearer than that of the others. Before giving the technic of the operation I will present the histories of two cases, to illustrate some of the points in diagnosis and the result of operation.

CASE I.—C. I., male, aged twenty-four years. Consulted me October 22, 1903, because he had lost a rubber drainage-tube into the antrum that he had been wearing in an opening through the tooth socket of his second molar. The antral trouble dates back three years, when he had a decayed tooth removed, part of the root remaining until about one year after the removal of the tooth, when he began to have a purulent discharge from the nose. He had the root extracted, a hole bored into the antrum through the socket, and a rubber drainage-tube inserted, which he wore until it slipped into the antrum six weeks ago. During the time he had the tube in place the discharge greatly diminished, but never entirely ceased. When I first saw him he was thin, anæmic, had gastric disturbances, headaches, pains about the eyes, a bad taste in the mouth, purulent discharge from the nose and alveolar opening, and nasal obstruction. On examining the nose, the membrane over the turbinates was swollen, middle more than the inferior, and pus was emerging from the middle meatus. Operated upon October 23. The antral mucous membrane was found in a state of polypoid degeneration from one-quarter to one-half of an inch in thickness, with the rubber tube in the cavity. No necrotic bone was found. The patient was out of bed the next morning, and the only evidence of the operation was some swelling of that side of the face, and he left the hospital the next day. November 13 the discharge had ceased, and he has gained fifteen pounds since the operation.

The preceding case is a clear one of antrum disease of dental origin. The following case shows the difficulty at times of arriving at a diagnosis and source of the trouble.

Case II.—W. Z. M., male, aged twenty-two years. Consulted me because of an offensive odor and purulent discharge from the nose, with severe headaches and vomiting. Two years previously he had an ulcerated tooth removed. He first noticed purulent discharge from the nose about three months later, but just previous to noticing the discharge he had his nasal septum operated upon and wore a nasal splint afterwards. Under these circumstances it is hard to say which was the cause of the infection, except that if it was caused by the nasal operation it probably would have subsided spontaneously. The patient himself thinks that the tooth caused the trouble. On examination, I find the affected side of the nose very narrow from the septum deviating to that side; the upper part of the nose filled with creamy pus, with an offensive odor of dead bone. The nose was so narrow and the mucous membrane so swollen that it was hard to make out the landmarks. There was a swelling of the mucous membrane in the region of the ethmoidal bulla and middle turbinate, with a perforation near the centre of this swelling leading into the antrum, and, on probing, dead bone was felt, but the pus coming from so high up in the nose led me to believe the trouble might be in the frontal sinus, but as that was the only symptom of frontal disease present I continued my investigation of the antrum, by puncturing the canine fossa and inflating the antrum, which caused the nose to fill with very offensive pus. Transillumination gave no aid, there not being much difference between the two sides. As I was sure of the antrum trouble, I operated upon that first. As a preliminary step to give me room in the nose, I straightened the septum. On entering the antrum I found the lining membrane from one-quarter to three-eighths of an inch in thickness, and necrosis of the upper portion of the nasal wall and of the ethmoid cells. The packing was removed on the fourth day and the nose douched thereafter.

The operation is performed under ether anæsthesia with the jaws held apart by a mouth-gag placed on the opposite side to the trouble, the choana on the side of the lesion tamponed, and a gauze packing placed between the cheek and the jaws well back to prevent blood from running into the pharynx. The cheek is retracted upward and outward with a blunt retractor placed in the angle of the mouth.

This gives a very good view of the field of operation. The incision is made from the last molar to the canine tooth, about one-quarter of an inch below the gingivo-labial fold, down to the bone. Then the periosteum is elevated over the entire anterior wall of the antrum, and as far on the lateral wall as necessary. Now, by placing the retractor into this incision and elevating the soft parts a good view is obtained of the canine fossa. With a chisel an opening is made at the most depressed portion of the fossa, large enough to admit a rongeur, with which the entire anterior and part of the lateral wall of the antrum are removed, care being exercised not to wound the membranous lining of the antrum, thereby avoiding troublesome hemorrhage. Now that the bony walls are removed, incise the lining mucous membrane and examine the interior of the antrum for granulations, character of membrane, foreign bodies, etc.

Remove the entire membranous lining of the antrum, giving especial care to the superior internal and external angles. The bone may now be examined for necrotic spots, which are more frequently found about the roots of the teeth, or on the upper nasal wall. Necrosis is frequent in the cases operated upon. It is almost needless to say that all necrotic bone must be completely removed, down to healthy tissue, and any partitions or ridges in the antrum should be broken down and made smooth with the walls. The next step is to remove the bony nasal wall completely, from the floor to the roof, from the anterior margin to the posterior. This is accomplished by placing the little finger into the nose, to act as a guide, and chipping the bone away with a curette or forceps. After a little practice this can be done quickly without injuring the mucous membrane. The inferior turbinate bone should also be shelled out in the same manner. The next step is to examine the ethmoid cells, and, if affected, they should be removed with a curette or forceps, and if the sphenoid sinus is involved, the anterior wall and lining membrane should be removed. Now, with the mucous membrane remaining on the nasal wall, flaps are made to help line the antrum. If you have all the membrane from the middle meatus to the floor, including that of the inferior turbinate, two large flaps can be made, one to partly cover the roof and external wall, and one for the floor. I divide the membrane from its anterior attachment and then make a horizontal incision just above the membrane of the inferior turbinate back to the attachment to the bone. The upper flap I place partly on the roof and partly on the external wall and suture the end to the periosteum at the external angle of the incision. The lower flap is laid on the floor of the antrum and sutured in a like manner to the periosteum, as far externally as it will reach.

I now pack the cavity firmly with iodoform gauze in such a way that it can be removed through the anterior naris, and then I close the buccal wound with silk sutures. On the fourth or fifth day the packing is removed and not replaced, also the sutures, the cavity simply being douched thereafter several times daily with a normal salt solution.

There is considerable swelling of the upper lip and cheek from the stretching received during the operation. Some cases need an opiate to relieve the pain after operation, as the infraorbital nerve is injured during the operation. There is no sensation in the soft parts for a number of weeks. The cases can generally leave the hospital the day after the packing is removed.

*Indications for the Radical Operation.*—If a diseased antrum has been treated for several months by one of the more conservative methods without result, the radical procedure becomes necessary to effect a cure. In certain cases, that have received no previous treatment, it seems useless to try the conservative operations,—namely, where there is considerable necrosis, which can generally be diagnosed by the odor and probe; or where the ethmoids and sphenoid sinus are extensively involved.

*Advantages of the Operation.*—You have a full view of the interior of the antrum, and in removing the nasal bony wall and using the nasal mucous membrane to partially line the antral cavity, the antrum, as such, is eradicated and made part of the nasal cavity proper, making a recurrence of the disease impossible, as there is no antrum, only a recess in the nose which is accessible through the anterior naris. The mucous membrane of the inferior turbinate continues to functionate, as is shown in an attack of acute coryza, when it swells, as does the turbinate of the other side. The opponents of this method contend that there are two disadvantages, —one, that it will cause a dry pharyngitis from increasing the caliber of the nasal chamber; in fact, it is not increased as much as they think, as the posterior third of the inferior turbinate remains, and part or all of the middle turbinate, unless the ethmoids have been involved. The other disadvantage, they contend, is that it does not always cure. I have yet to see a case it has not cured where the disease has been fearlessly and thoroughly removed.

